# Symptomatic primary hyperparathyroidism in a young woman presenting with multiple skeletal destructions: a case report and review of literature

**DOI:** 10.1186/s12902-020-00669-3

**Published:** 2021-01-07

**Authors:** Shuai Lu, Maoqi Gong, Yejun Zha, Aimin Cui, Wei Tian, Xieyuan Jiang

**Affiliations:** 1 Department of Orthopedic Trauma, Beijing Jishuitan Hospital, Peking University Fourth School of Clinical Medicine, Beijing, 100035 China; 2 Department of General Surgery, Beijing Jishuitan Hospital, Peking University Fourth School of Clinical Medicine, Beijing, China; 3 Department of Spine Surgery, Beijing Jishuitan Hospital, Peking University Fourth School of Clinical Medicine, No.31 Xinjiekou E Rd, Xicheng District, Beijing, 100035 China

**Keywords:** Hyperparathyroidism, Multiple skeletal destructions, Case report

## Abstract

**Background:**

Multiple pathological manifestations are rarely present in patients with primary hyperparathyroidism (PHPT). Here we described a case of a young woman who presented with multiple skeletal destructions and received an unclear diagnosis at several hospitals.

**Case presentation:**

A 30-year-old woman was admitted to our hospital due to pain in both knees and walking difficulty that lasted for 6 and 2 years, respectively. Her laboratory test results revealed a high parathyroid hormone level (822 pg/ml) and hypercalcemia (2.52 mmol/L) in the blood. Parathyroid imaging revealed a lumpy concentration of radioactive uptake detected at the lower pole in the right lobe of the thyroid, and was nearly 2.2 cm * 2.4 cm in size. Next, the patient was treated with parathyroidectomy that resulted in a significant improvement in physiological and clinical symptoms. Moreover, the skeletal destruction and bone mineral density were significantly improved after a 5-years follow-up period.

**Conclusions:**

Multiple skeletal destructions can be caused by PHPT that should be taken into consideration in young patients with complex bone lesions.

## Background

Primary hyperparathyroidism (PHPT) is a rare clinical condition characterized by an autonomic oversecretion of parathyroid hormone caused by a parathyroid adenoma, hyperplasia, or cancer [1, 2]. Hypercalcemia and hypophosphatemia have been observed in patients diagnosed with PHPT. These conditions are involved in multiple pathological manifestations in the whole body and are mainly detected in bone and urinary tract systems [3]. The prevalence of PHPT has been reported to be 1% across the world, while the prevalence in China is nearly 5%. A previous study found that the prevalence of PHPT co-existence with osteoporosis significantly increased with the increase of age [4]. The decrease in bone mineral density was mainly detected in the areas with a high proportion of cortical layers, and the risk of fracture was significantly increased in all parts of the bone. Moreover, nervous, digestive, urinary, and other systems were also affected in patients with PHPT [5]. In western countries, PHPT is more commonly detected at early stages due to the comprehensive screening available in those countries. Therefore, most of the PHPT cases are of asymptomatic type, with rare cases of bone destruction [6]. However, the prevalence of PHPT in China is high, and most cases presented are symptomatic [7]. Therefore, it is challenging to establish a clear diagnosis at an early stage, which results in high misdiagnosis rates in China [8]. Consequently, we described a case of a young woman who presented with multiple skeletal destructions and received an unclear diagnosis at several hospitals.

## Case presentation

**Fig. 1:**
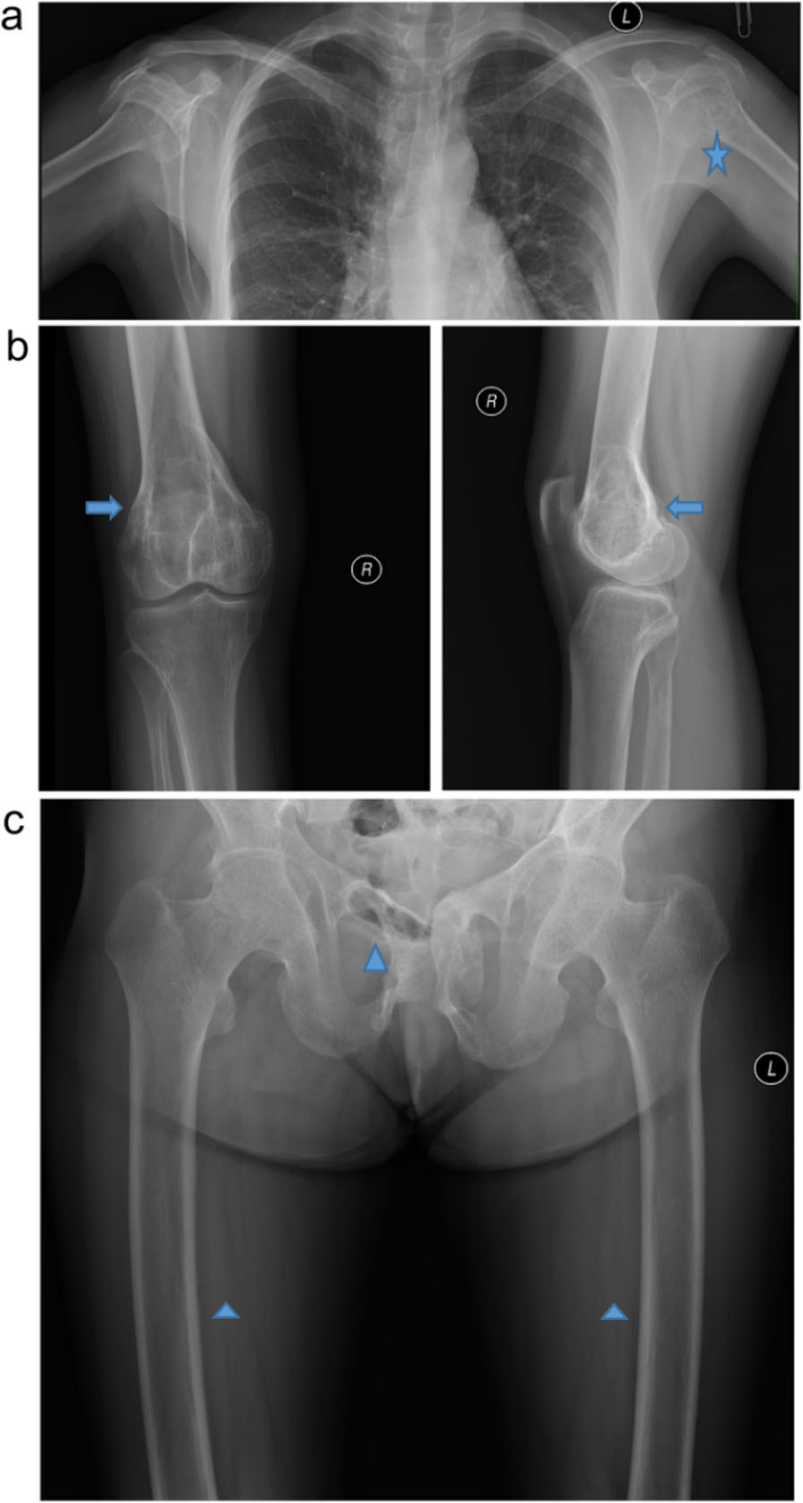
The films reveal bone lesions at different points of the patient. **a** The star highlights old healing of the fracture, thinning of the bone cortex, and multiple cystic lesions of the shoulder. **b** The arrows mark old fracture and multiple cystic lesions of the distal femur. **c** The triangles highlight old healing of the hip fracture and both lower limbs malunion

A 30-year-old woman was admitted to Beijing Jishuitan Hospital on 21 May, 2014, due to pain in both knees and walking difficulty that lasted for 6 and 2 years, respectively. Six years ago, the patient presented with pain in both knees and hips without obvious inducement, and the pain was aggravated following movement, flexion, and extension. The patient received no specific treatment. Nearly 2 years ago, the pain in the knee became worse. She reported a right distal femoral fracture when enduring a mild external force (Fig. 1), for which she was given conservative treatment. An additional fracture at the right hip and left shoulder occurred due to mild external force 22 months ago. About 1 year ago, her lower limbs gradually tapered, bended, and deformed, resulting in the height reduction from 163 cm to 159 cm. Next, the patient was admitted to a local hospital and was suspected of having a malignant bone tumor. After this, she was admitted into the General Hospital of PLA, and puncture was conducted at the distal of the right femur. The biopsy results suggested a potential giant cell tumor, for which the patient was treated with cisplatin chemotherapy at the local hospital. The bone pain was not relieved following the treatment, and it gradually worsened after 2 cycles of chemotherapy.

The patient was admitted to the department of bone oncology at Beijing Jishuitan Hospital 1 month ago. Her urinary calculi and hypertension history lasted for 6 and 3 years, respectively. The results of physical examination revealed good lower limb symmetry, similar length; “O” sharp. Moreover, the muscle atrophy was obvious in lower limbs, and flexion or extension of hip and knee joints were restricted.

The results of the biochemical examination found the parathyroid hormone, calcium, and phosphorus in the blood were 822 pg/ml, 2.52 mmol/L, and 0.42 mmol/L, respectively. Parathyroid imaging revealed a lumpy concentration of radioactive uptake at the lower pole in the right lobe of thyroid that was nearly 2.2 cm * 2.4 cm in size. The results of dual-energy X-ray absorption assay found the T at L1-L4 was − 4.7, and T at the femoral neck was − 4.0. The patient was suspected with a metabolic bone disease caused by PHPT, after which she was transferred to the department of general surgery for further PHPT treatment. The surgical contraindications were excluded through relevant examinations after admission. Right parathyroid gland resection was conducted after 6 days, and the results of intraoperative freezing and postoperative pathological suggested that the patient had parathyroid adenoma. Numbness around the mouth was observed 2 days postoperatively and were treated with calcium gluconate, after which the symptoms were relieved 3 days postoperatively. All biochemical parameters were improved, and the bone pain was significantly relieved 1 week postoperatively.

The patient was re-examined after 5.0 years. Her biochemical examination results revealed that parathyroid hormone, calcium, and phosphorus in the blood were returned to normal with 56 pg/ml, 2.3 mmol/L, 0.9 mmol/L, respectively. Moreover, the bone mineral density was significantly increased compared with the preoperative condition with the T at L1-L4 reaching − 1.11, and T at femoral neck 0.1. Finally, the prevalence of muscle and bone pain was significantly reduced, and patient’s life quality was significantly improved.

## Discussion and conclusion

This is a relatively rare case of multiple skeletal destructions in addition to PHPT. This patient had longer disease duration and continuous bone destruction due to misdiagnosis. She already presented with indicative bone manifestations visible in imaging scans, pathological fractures, and bone deformities. Therefore, the potential pathological mechanisms underlying bone destruction in PHPT patients should be further explored. In this study, the parathyroid hormone, calcium, phosphorus in the blood, bone mineral density, and relevant symptoms significantly improved after parathyroidectomy. Therefore, for improving the diagnosis of PHPT, it is worth investigating whether multiple skeletal destruction or PHPT exists independently.

The main clinical manifestations in this patient were a pain in both knees and walking difficulty. Moreover, fracture at the distal right femur, right hip, and left shoulder was further detected after suffering mild external force. PHPT exerts a critical role in the skeletal system through the regulation of parathyroid hormone and calcium ion. The release of calcium and phosphorus in the bone matrix is significantly increased when stimulated by parathyroid hormone, thus increasing extracellular calcium ion concentration. Moreover, the potential impacts of PHPT on the skeletal system could be divided into catabolic and anabolic, which depend on whether the raising of parathyroid hormone has transient or long-term duration [9]. The presence of long-term elevated parathyroid hormone yields a greater role in bone resorption than remodeling. Moreover, the parathyroid hormone in peripheral circulation was significantly increased, causing a significant decrease in bone mineral density and systemic metabolic bone changes.

The bone destruction was accumulated, and the fractures, skeletal deformities were detected if a patient with symptomatic PHPT could not be diagnosed at an early stage. The above clinical symptoms are significantly correlated with bone mineral density, bone brittleness, and bone trabecular structure. In patients suffering from PHPT over a long-term period, it always coexists with severe vitamin D deficiency, which could have devastating consequences on the skeleton [10–12]. In the present study, the T value and Z value of bone mineral density measured by dual-energy x-ray absorptiometry (DXA) in the lumbar spine and femoral neck were significantly decreased. A previous study already reported that bone mineral density was mainly decreased in areas with a higher proportion of cortical bone (femoral neck and distal radius), while the lower decrease was observed in the vertebral body with more cancellous bone [13]. However, in the case, lumbar bone density was worse than cortical bone. We hypothesize that this phenomenon results from the long-term effects of severely elevated PTH on the skeletal system. The elevated PTH involves bone system and presents abnormal bone metabolism, multi-site fracture, bone cyst lesion, bone malunion, and so on. In the following clinical practice, if similar cases are encountered, the causes of this phenomenon need to be further summarized and analyzed. Moreover, bone mineral density could predict further fracture and could be used as an essential indication for further surgery [14]. Therefore, the high proportion of cortex was associated with greater bone mineral density loss, resulting in a higher risk of fracture. However, the fracture at vertebral and other sites are significantly increased in patients with PHPT [15]. Recently, another study reported abnormal bone system trabecular microstructures and decreased bone hardness at an early stage due to the application of high-resolution peripheral quantitative computed tomography [16], which could explain why the vertebral and non-vertebral fractures risk are both increased in PHPT patients.

PHPT patients in China mainly present with bone parathyroidism, and severe bone destruction. The potential reason for this could be a misdiagnosis and delayed treatment. PHPT patients are commonly diagnosed with rheumatoid arthritis, osteoarthrosis, traumatic fractures, bone tuberculosis, and multiple myeloma. Therefore, patients with unknown cause of bone destruction should undergo the biochemical examination for parathyroid hormone, calcium, and phosphorus. Moreover, surgery is a definitive treatment for PHPT, and the bone mineral density and clinical symptoms tend to significantly improve after parathyroidectomy. However, the hungry bone syndrome might occur because of the rapid accrual of calcium from the intravascular compartment into the skeleton [17, 18]. Finally, the bone resorption was significantly reduced after parathyroidectomy, while bone formation could be further affected by secondary serum parathyroid hormone [19, 20]. At the same time, some researchers reveal that musculoskeletal findings are still a important presentation in patients with primary hyperparathyroidism. Some of these manifestations can be quite unusual and may represent diagnostic dilemmas [21]. Daniela Gallo, et al. reveal a patient keeps of brown tumors and misdiagnosed as bone cancer. In their opinion, it is important to discuss the differential diagnosis in uncommon bone disorders. And the multidisciplinary approach involving pathologist, endocrinologist and oncologist should be created in the diagnostic and therapeutic work-up [22]. Our hospital is dominated by symptomatic patients, and this population can then be explored for a single-center study in the future.

The multiple manifestations in the skeletal system are found in patients with PHPT, and the symptomatic PHPT patient could also present with multiple system symptoms that could result in the high misdiagnosis rate. The results of this study could be used to guide further diagnosing of PHPT at the early stage.

## Data Availability

The datasets used and/or analyzed during the current study are available from the corresponding author on reasonable request.

## Abbreviations

SPHPT: Symptomatic primary hyperparathyroidism
BMD: Bone mineral density
PHPT: Primary hyperparathyroidism
DXA: Dual energy x ray absorptiometry
